# Widespread *Wolbachia* infection in terrestrial isopods and other crustaceans

**DOI:** 10.3897/zookeys.176.2284

**Published:** 2012-03-20

**Authors:** Richard Cordaux, Samuel Pichon, Houda Ben Afia Hatira, Vincent Doublet, Pierre Grève, Isabelle Marcadé, Christine Braquart-Varnier, Catherine Souty-Grosset, Faouzia Charfi-Cheikhrouha, Didier Bouchon

**Affiliations:** 1 Université de Poitiers, CNRS UMR 6556, Laboratoire Ecologie Evolution Symbiose, 40 Avenue du Recteur Pineau, 86022 Poitiers, France; 2 Faculté des Sciences de Tunis, Unité de Recherche de Bioécologie et Systématique Evolutive, 2092 Université Tunis Manar, Tunisia; 3 Present address: Zoological Institute, University of Basel, Vesalgasse 1, 4051 Basel, Switzerland; 4 Present address: Institut für Biologie, Martin-Luther-Universität Halle-Wittenberg, Hoher Weg 8, 06120 Halle (Saale), Germany

**Keywords:** *Wolbachia*, endosymbiont, Crustacea, Maxillopoda, terrestrial isopod, distribution, adaptation

## Abstract

*Wolbachia* bacteria are obligate intracellular alpha-Proteobacteria of arthropods and nematodes. Although widespread among isopod crustaceans, they have seldom been found in non-isopod crustacean species. Here, we report *Wolbachia* infection in fourteen new crustacean species. Our results extend the range of *Wolbachia* infections in terrestrial isopods and amphipods (class Malacostraca). We report the occurrence of two different *Wolbachia* strains in two host species (a terrestrial isopod and an amphipod). Moreover, the discovery of *Wolbachia* in the goose barnacle *Lepas anatifera* (subclass Thecostraca) establishes *Wolbachia* infection in class Maxillopoda. The new bacterial strains are closely related to B-supergroup *Wolbachia* strains previously reported from crustacean hosts. Our results suggest that *Wolbachia* infection may be much more widespread in crustaceans than previously thought. The presence of related *Wolbachia* strains in highly divergent crustacean hosts suggests that *Wolbachia* endosymbionts can naturally adapt to a wide range of crustacean hosts. Given the ability of isopod *Wolbachia* strains to induce feminization of genetic males or cytoplasmic incompatibility, we speculate that manipulation of crustacean-borne *Wolbachia* bacteria might represent potential tools for controlling crustacean species of commercial interest and crustacean or insect disease vectors.

## Introduction

*Wolbachia pipientis* (hereafter *Wolbachia*) bacteria are obligate intracellular alpha-Proteobacteria of arthropods and nematodes (O’Neill et al. 1997; Bourtzis and Miller 2003). These maternally-inherited bacteria are often referred to as reproductive parasites because they are able to manipulate the reproduction of their hosts to increase their own transmission, via mechanisms such as cytoplasmic incompatibility, male killing, thelytokous parthenogenesis and feminization of genetic males (O’Neill et al. 1997; Bourtzis and Miller 2003; Cordaux et al. 2011). In addition to vertical transmission, *Wolbachia* bacteria are occasionally transmitted horizontally (Werren et al. 1995; Vavre et al. 1999; Cordaux et al. 2001). These transmission patterns probably explain, at least partly, why *Wolbachia* bacteria are found in a highly diverse range of hosts.

*Wolbachia* bacteria are particularly frequent in arthropods. Hence, it has been estimated that 20–75% of insect species may be infected by *Wolbachia* (Werren and Windsor 2000; Hilgenboecker et al. 2008). These bacteria have also been reported in chelicerates such as mites (Breeuwer and Jacobs 1996), spiders (Cordaux et al. 2001) and scorpions (Baldo et al. 2007). In crustaceans, *Wolbachia* have long been known to infect the terrestrial isopod *Armadillidium vulgare* (Rousset et al. 1992) (order Isopoda, suborder Oniscidea; classification from Martin and Davis (2001)), in which *Wolbachia* induce functional feminization of genetic males (Rigaud et al. 1997; Cordaux et al. 2004; Bouchon et al. 2008; Cordaux et al. 2011). A systematic search in 80 crustacean species suggested that *Wolbachia* infection was restricted to isopods, with a prevalence of 46% in terrestrial isopod species (Bouchon et al. 1998). Based on additional screenings, this figure has recently been updated to 61% (Bouchon et al. 2008). The initial screening of 80 crustacean species also provided molecular evidence for *Wolbachia* infection in two other isopod suborders (Asellotta and Flabellifera) (Bouchon et al. 1998).

Despite multiple screenings of crustacean groups, *Wolbachia* bacteria have seldom been found in non-isopod crustacean species (Bouchon et al. 1998; Fitzsimmons and Innes 2005; Maniatsi et al. 2010). To date, only two amphipod and two non-marine ostracod crustacean species outside of the Isopoda order have been reported to be infected by *Wolbachia* (Cordaux et al. 2001; Baltanas et al. 2007). The extent to which this uneven *Wolbachia* distribution among crustacean species reflects host spectrum specificity or biased sampling remains to be clarified. In this study, we report *Wolbachia* infections in fourteen new crustacean species. Our results extend the range of *Wolbachia* infection within terrestrial isopods and amphipods (class Malacostraca). Moreover, the discovery of *Wolbachia* in the goose barnacle *Lepas anatifera* (subclass Thecostraca) establishes *Wolbachia* infection in class Maxillopoda. Molecular characterization indicates that the new strains are closely related to B-supergroup *Wolbachia* strains previously reported from crustacean hosts (Bouchon et al. 1998; Cordaux et al. 2001). The identification of closely related *Wolbachia* strains in highly divergent crustacean hosts suggests that this group of *Wolbachia* endosymbionts can naturally adapt to a wide range of crustacean hosts, not just isopods.

## Materials and methods

Wild-caught individuals belonging to fourteen crustacean species were studied (Table 1). Seven species have been previously detected in a survey of *Wolbachia* infection of woodlice fauna in Tunisia (Ben Afia Hatira et al. 2007). For these species, *Wolbachia* prevalence (ranging from 40% to 100%) is available in the original publication. The seven remaining species were included as part of an ongoing effort in our laboratory to sample and test novel crustacean species for *Wolbachia* infection. For these species, one or two individuals were collected. Therefore, no information on prevalence is available for these species. Genomic DNA from single individuals was extracted as previously described (Bouchon et al. 1998). *Wolbachia* infection status of each individual was tested using a PCR assay based on the standard *wsp* marker. We used primers 81f and 691r (Braig et al. 1998) and previously described PCR conditions (Cordaux et al. 2001). Purified PCR products were directly sequenced in both directions, as previously described (Cordaux et al. 2001). The *wsp* sequences generated in this study were deposited in GenBank under accession numbers HE616815-HE616830 (Table 1).

**Table 1.** Novel crustacean species infected by *Wolbachia* bacteria reported in this study.

**Class**	**Infraclass (I) or Order (O)**	**Species**	**Sampling location**	**GenBank accession number for *ws*p**
Maxillopoda	Cirripedia (I)	*Lepas anatifera*	La Rochelle, France	HE616817
Malacostraca	Amphipoda (O)	*Talitrus saltator*	La Rochelle, France	HE616820 and HE616821
	Isopoda (O)	*Armadillidium granulatum*	Sidi Massaoud Khniss, Tunisia	HE616828
		*Armadillidium pelagicum*	Ras Jbel, Tunisia	HE616829
		*Armadillidium sulcatum*	Natural Reserve Mhibes, Tunisia	HE616827
		*Cubaris murina*	Baie Mahault, Guadeloupe, France	HE616815
		*Hemilepistus reaumuri*	Metbasta, Tunisia	HE616816
		*Platyarthrus hoffmansegghi*	Liniers, France	HE616818
		*Porcellio albinus*	Kibili, Tunisia	HE616830
		*Porcellio buddelundi*	Skhira cliff, Tunisia	HE616823 and HE616824
		*Porcellio lamellatus*	Menzel Jmil, Tunisia	HE616825
		*Porcellio variabilis*	Jbel Ouest, Tunisia	HE616826
		*Porcellionides cingendus*	Archigny, France	HE616819
		*Trachelipus rathkei*	Cosne Cours sur Loire, France	HE616822

Sequences were aligned using ClustalW as implemented in the software BioEdit ver. 7.0 (Hall 1999), followed by manual adjustments. Representative sequences from the B supergroup of *Wolbachia* diversity were included for comparison and two of the A supergroup as an outgroup, as previously described (Cordaux et al. 2001; Cordaux et al. 2004). There was a total of 618 positions in the dataset. Hypervariable regions were deleted because they could not be aligned with confidence. The resulting alignment included 512 positions of which 185 were considered informative by parsimony criteria. Recombination analyses were performed using Recombination Detection Program (RDP) v3.41 (Martin et al. 2010). Parameters were set as follows: sequences were considered linear, the highest acceptable P value cutoff was 0.01, a Bonferroni correction was applied, consensus daughter sequences were found, gaps were included, different window sizes of variable sites were tested (10, 20, and 30 VI), and 1,000 permutations were performed. Phylogenetic analyses were conducted using the Minimum Evolution (ME) and Neighbor-Joining (NJ) methods, as implemented in MEGA ver 4.0 (Tamura et al. 2007). Evolutionary distances were computed using the Kimura 2-parameter substitution model. The ME tree was searched using the close-neighbor-interchange algorithm at a search level of 1 and the NJ algorithm was used to generate the initial tree. All positions containing alignment gaps and missing data were eliminated in pairwise sequence comparisons (pairwise deletion option). Bootstrap analyses were carried out with 1000 replicates.

## Results and discussion

Our findings extend the range of *Wolbachia* infections among crustacean hosts to twelve additional terrestrial isopod species, one amphipod species and one cirriped species (class Maxillopoda) (Table 1). We performed a molecular characterization of the *Wolbachia* strains based on the *wsp* gene, a *Wolbachia*-specific genetic marker (Braig et al. 1998) which has been shown to be highly informative for the analysis of crustacean *Wolbachia* strains (Cordaux et al. 2001). In total, we identified sixteen different *Wolbachia* strains: two strains in the amphipod *Talitrus saltator* and the terrestrial isopod *Porcellio buddelundi*, and one strain in each of the other crustacean host species. This is the third report of multiple *Wolbachia* strains being harbored by a single terrestrial isopod host species and the first case reported in amphipods. Indeed three different *Wolbachia* strains have previously been identified in *Armadillidium vulgare* (Cordaux et al. 2004; Verne et al. 2007) and *Porcellionides pruinosus* (Michel-Salzat et al. 2001). As in *Armadillidium vulgare* and *Porcellionides pruinosus*, the *Wolbachia* strains found in *Talitrus saltator* and *Porcellio buddelundi* were identified in different individuals, and no multiple infection of single individuals have been reported so far in crustaceans, in contrast to what has been widely documented in insects (Vautrin et al. 2007).

Querying GenBank with the sixteen *wsp* sequences through BLASTN searches resulted in best matches to other *Wolbachia* strains with 98.0–100% nucleotide similarity. This analysis revealed that all novel *Wolbachia* strains belong to the B supergroup of *Wolbachia* diversity, as do all isopod and amphipod *Wolbachia* strains characterized to date (Rousset et al. 1992; Bouchon et al. 1998; Cordaux et al. 2001; Michel-Salzat et al. 2001; Cordaux et al. 2004).

**Figure 1.**
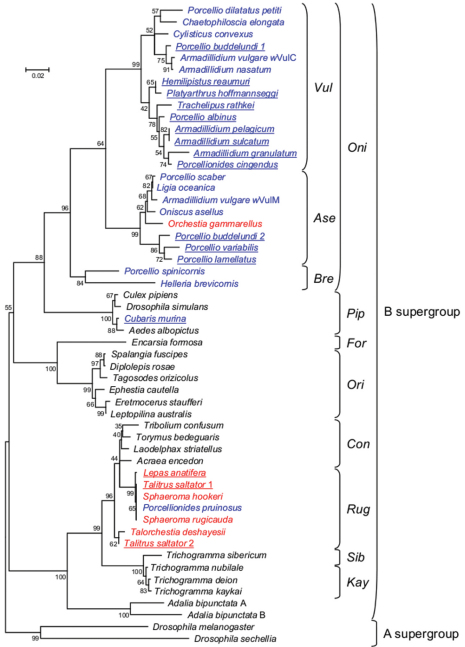
Phylogenetic tree of B-supergroup *Wolbachia* strains based on *wsp* sequences, using Minimum Evolution analysis. The tree is rooted with two A-supergroup *Wolbachia* strains. Bootstrap values inferred from 1000 replicates are shown as percentages. Strains are identified by the host species from which they were isolated. *Wolbachia* strains from terrestrial isopods and non terrestrial isopod crustaceans are shown in blue and red, respectively. New crustacean *Wolbachia* infections reported in this study are underlined. *Wolbachia* strains from insects are shown in black. Names assigned to groups of *Wolbachia* strains are shown on the right, following Cordaux et al. (2001).

To further characterize the novel crustacean *Wolbachia* strains, we performed a phylogenetic analysis of B supergroup *Wolbachia* strains. To facilitate comparisons with previous analyses of crustacean *Wolbachia* strains, we added the sixteen strains reported in this study to the set of representative B supergroup strains previously used in Cordaux et al. (2001). No significant support for recombination was detected with RDP and the topologies of the ME and NJ trees (see Materials and Methods) reconstructed in this study were highly similar, as they were to the phylogenetic inferences reported previously (Cordaux et al. 2001). We also emphasize that, as far as crustacean *Wolbachia* strains are concerned, we have shown previously that a *wsp*-based phylogeny yielded essentially the same relationships between taxa as phylogenies based on other *Wolbachia* genes such as *16S rRNA*, *ftsZ* and *GroE* (Bouchon et al. 1998; Cordaux et al. 2001; Wiwatanaratanabutr et al. 2009). Because our updated phylogeny of crustacean *Wolbachia* strains is in agreement with phylogenetic results obtained in previous studies using various genes, we are reasonably confident that the phylogenetic analysis we present in the manuscript is reliable. Figure 1 shows the tree inferred from the ME analysis. All but one crustacean *Wolbachia* strains clustered together in one of the two groups *Oni* and *Rug*, previously shown to encompass isopod and amphipod *Wolbachia* strains (Bouchon et al. 1998; Cordaux et al. 2001; Wiwatanaratanabutr et al. 2009). Our extended dataset shows a newly emerging trend in the diversity of crustacean *Wolbachia* strains, in that the *Oni* group mostly contains strains isolated from terrestrial isopods, whereas the *Rug* group mostly contains strains isolated from non terrestrial isopod crustaceans. We speculate that this might reflect two major ancestral *Wolbachia* acquisitions in crustaceans, in terrestrial and aquatic environments. Ecosystems in the transition zones from land to sea, as estuaries and wetlands, are highly productive and therefore attract a multitude of wildlife. Moreover these ecosystems are in a continuous state of change. The finding of closely related symbionts in the *Rug* group suggesting recent symbiont dispersal across divergent hosts sharing the same habitats suggests that horizontal transfers of *Wolbachia* could be facilitated in such ecosystems.

The only exception to the clustering of crustacean *Wolbachia* strains in the *Oni* or *Rug* groups is the *Wolbachia* strain isolated from the terrestrial isopod *Cubaris murina* (Fig. 1). Indeed, the latter strain falls within the *Pip* group, closely related to *Wolbachia* strains from mosquitoes and drosophila. This result suggests the possibility of a *Wolbachia* horizontal transfer between isopod and insect hosts, as previously proposed to explain the similarity between strains of the *Rug* group with other insect *Wolbachia* strains (Bouchon et al. 1998; Cordaux et al. 2001). In any event, it is noteworthy that *Cubaris murina* is a terrestrial isopod with a pan-tropical distribution (Schmalfuss 2003). Interestingly, a similar result has been obtained by Wiwatanaratanabutr et al. (2009) who showed that the *Wolbachia* strain isolated from an endemic isopod sampled in Thailand also belongs to the *Pip* group. This is in contrast to most terrestrial isopods screened so far for *Wolbachia* infection, which were generally sampled in temperate regions (Bouchon et al. 1998; Bouchon et al. 2008). This suggests that screenings of terrestrial isopods and other crustaceans in regions that have not been extensively studied so far may have the potential to uncover a tremendous and unexpected *Wolbachia* diversity that we are only beginning to realize.

An important result of this study is the discovery of *Wolbachia* in the goose barnacle *Lepas anatifera*. This is the first report of *Wolbachia* infection in the class Maxillopoda, a major crustacean group comprising ~15,000 species, that is, more than one quarter of all described crustacean species (Martin and Davis 2001). Interestingly, the Maxillopoda *Wolbachia* strain is closely related to other crustacean *Wolbachia* strains in the *Rug* group, being 99.8% similar to the *Wolbachia* strains from the terrestrial isopod *Porcellionides pruinosus*, the intertidal isopods *Sphaeroma rugicauda* and *Sphaeroma hookeri* and the amphipod *Talitrus saltator*, based on the *wsp* marker (Fig. 1). This result thus indicates that highly divergent crustacean hosts can harbour highly similar *Wolbachia* endosymbionts. By implication, our results suggest that this group of *Wolbachia* endosymbionts can naturally adapt to a wide range of crustacean hosts. Given the demonstrated ability of terrestrial isopod *Wolbachia* strains to induce feminization of genetic males or cytoplasmic incompatibility between infected males and uninfected females (Legrand and Juchault 1986; Rigaud et al. 1997; Bouchon et al. 1998; Moret et al. 2001; Cordaux et al. 2004; Bouchon et al. 2008; Cordaux et al. 2011), we speculate that manipulation of crustacean-borne *Wolbachia* bacteria might represent potential tools for controlling crustacean species of commercial interest and crustacean or insect disease vectors. For example, it has been shown that freshwater crustaceans could be used as predators to control immature forms of the mosquito *Aedes aegypti*, the vector of the Dengue fever, one of the major infectious diseases in several tropical and subtropical countries in Asia, Africa, and The Americas (Kosiyachinda et al. 2003). The finding of *Wolbachia* infection in a wide range of crustacean species, including freshwater crustaceans, may open new opportunities in the biological control of insect disease vectors via a *Wolbachia*-based strategy. This might allow researchers to manipulate the population dynamics of crustacean predators of insects, to enhance their efficacy as biological control agents.
